# An Unusual Case of Apixaban-Induced Small Vessel Vasculitis: Leukocytoclastic Vasculitis

**DOI:** 10.7759/cureus.55040

**Published:** 2024-02-27

**Authors:** Abigail Y Adebayo, Azka Ali, Roshan M Goswami, Oluwatosin Abimbolu, Khudija Nayab, Henry Onyemarim

**Affiliations:** 1 Emergency Medicine, Glangwili General Hospital, Wales, GBR; 2 Internal Medicine, Rosalind Franklin University of Medicine and Science, North Chicago, USA; 3 Internal Medicine, American University of Antigua, Antigua, ATG; 4 Family Medicine, St Helen’s and Knowsley NHS Trust, Coventry, GBR; 5 Internal Medicine, Khyber Girls Medical College, Peshawar, PAK; 6 Pathology and Laboratory Medicine, Asaba Specialist Hospital, Asaba, NGA

**Keywords:** direct oral anticoagulant side effect, lcv, apixaban induced leukocytoclastic vasculitis, leukocytoclastic vasculitis (lcv), drug-induced hypersensitivity

## Abstract

Apixaban is a rare cause of drug-induced leukocytoclastic vasculitis (LCV). We report a case of apixaban-induced LCV in a 55-year-old male with deep vein thrombosis who developed systemic symptoms and pruritic rash in the bilateral lower extremity after 17 days of apixaban therapy. A skin biopsy confirmed the LCV, and he was diagnosed with apixaban-induced LCV after ruling out all other possible causes. His condition improved after apixaban discontinuation, supportive management, and oral prednisone. Our case highlights the early diagnosis and management of drug-induced LCV and also describes the existing literature to highlight existing knowledge and potential mechanisms underlying anticoagulant-induced vasculitis.

## Introduction

Apixaban is a novel oral anticoagulant and reversible direct inhibitor of factor Xa, which is primarily indicated for prophylaxis and management of systemic thromboembolism and non-valvular atrial fibrillation (AF). Apixaban is also prescribed for the prevention of stroke and venous thromboembolism [1]. Apixaban is generally a well-tolerated drug; however, certain clinical side effects have been reported. Bleeding (major or non-major) is the most reported side effect of apixaban. Other less-reported side effects include nausea, anemia, hematuria, hematoma, or elevated transaminases [2]. Hypersensitivity reaction due to apixaban is a rare and life-threatening adverse event, and only a few cases have been reported [3]. We report a case of drug-induced leukocytoclastic vasculitis (LCV) as a hypersensitivity reaction to apixaban use.

## Case presentation

A 55-year-old male presented with mild fever, arthralgia, and lower extremity rash for the last three days. The pain was gradual in onset, progressive over time, worse on movement, and relieved by taking analgesics. The fever was mild, intermittent, and relieved by taking paracetamol. The rash was bilateral and first appeared on his feet, followed by involvement of the legs and thighs. He had a history of hypertension, congestive heart failure, coronary artery disease, and deep vein thrombosis with a body mass index of 26. His medication included aspirin, furosemide, glyceryl nitrate, apixaban, atorvastatin, and losartan. Recently, he was commenced on apixaban, and warfarin was withheld 17 days ago due to challenges associated with monitoring requirements of warfarin due to financial constraints. He had no history of drug allergy, chemical exposure, or insect bite. He reported no mucosal bleeding, melena, or hematochezia.

**Figure 1:**
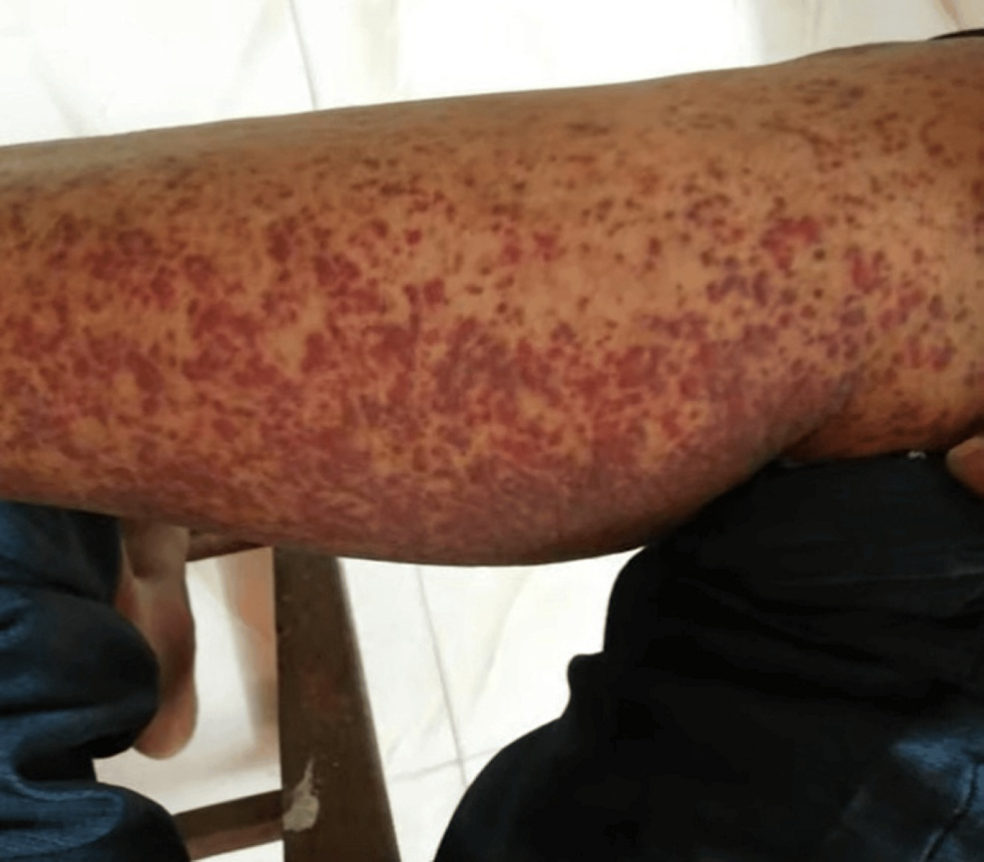
Skin showing well-defined lesions of varying sizes, displaying a spectrum of colors from dark red to violet

On examination, he was hemodynamically stable with a blood pressure of 140/90 mmHg and a temperature of 99^o^F. On skin examination, there was widespread, mildly tender, non-itchy palpable purpura on his lower extremities, such as feet, legs, and thighs. The lesions were well-defined and of varying sizes, displaying a spectrum of colors from dark red to violet (Figure 1). Respiratory and cardiovascular systems examinations were unremarkable.

**Figure 2:**
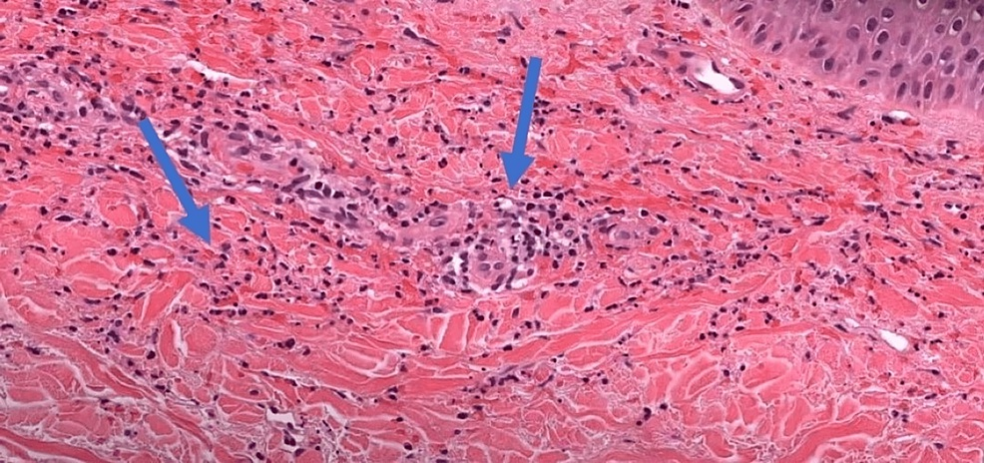
Biopsy of lesion demonstrating abundant scattered neutrophils and fibrinoid necrosis of vessel wall (blue arrows) Eosin and hematoxylin stain (magnification: 40x).

He underwent comprehensive serological and biochemical testing to rule out possible etiology, which revealed mild thrombocytopenia, elevated erythrocyte sedimentation rate, and low complement level (Table 1). A biopsy of the lesion revealed neutrophilic infiltration in the superficial perivascular region, coupled with melanophages and eosinophils with leukocytoclastic debris, consistent with LCV (Figure 2). Further workups, including blood culture, hepatitis screening, human immunodeficiency virus (HIV) screening, urine analysis, syphilis screening, and autoimmune screening, were unremarkable.

**Table 1:** Results of laboratory evaluations ANA: Antineutrophil cytoplasmic antibody.

Parameters	Lab value	Reference range
Red cell count	3.2	4.20-5.65 million cells/ul
White cell count	7,200	4,000-11,000 cells/mm^3^
Neutrophil count	58%	40%-60%
Lymphocyte count	34%	20%-40%
Monocyte count	7%	6%-8%
Eosinophil count	1%	1%-4%
Hemoglobin	8.9	13-16 g/dl
Platelet count	71,000	150,000-350,000 cells/mm^3^
Blood urea nitrogen	29	13-41 mg/dl
Serum creatinine	0.9	0.6-1.2 mg/dl
Alanine aminotransferase	50	0-55 IU/L
Aspartate aminotransferase	34	0-37 IU/L
Erythrocyte sedimentation rate	31	0-22 mm/hr
Complement C4 level	18	15-53 mg/dl
Complement C3 level	101	82-185 mg/dl
ANA screening	Negative	
Rheumatoid factor	Negative	
C-reactive protein	1.1	0.3-1.3 mg/dl
Cryoglobulin	Negative	0-30 IU/ml

He was diagnosed with LCV due to apixaban after ruling out all other possible causes. He was managed with oral prednisone 40 mg/daily for two months with a tapering dose. Apixaban was replaced with dabigatran 110 mg twice daily. His rash rapidly improved with the normalization of platelet count and inflammatory markers.

## Discussion

Apixaban is a novel direct oral anticoagulant used for the treatment and prevention of cardiovascular disease, and side effects reported after apixaban use are tabulated in Table 2 [4].

**Table 2:** Reported side effects of apixaban use in patients with deep vein thrombosis Source: Ref. [4].

Side effect	Number	Frequency
Bleeding	12	1.8
Headache	17	2.5
Abdominal pain	16	2.4
Nausea	6	0.89
Rash/hypersensitivity	3	0.45
Itching	5	0.75
Paresthesia	2	0.30

**Figure 3:**
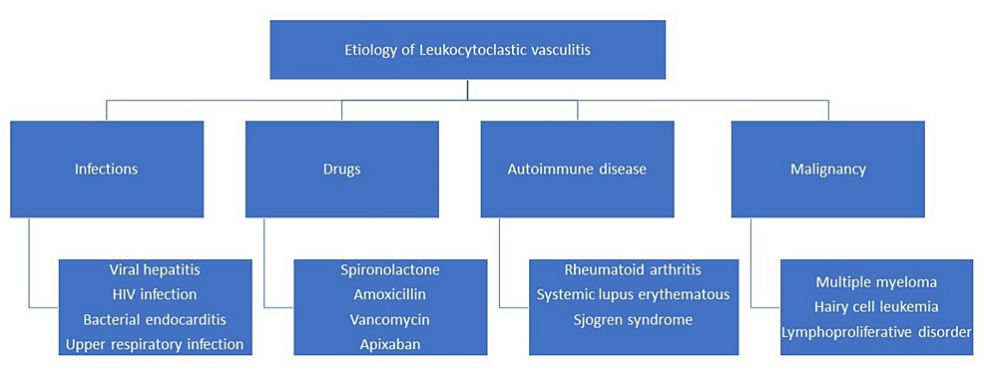
Etiology of leukocytoclastic vasculitis. Image credit: This image was created by one of the authors, Azka Ali.

LCV is an inflammatory disorder of small vessels of the skin, characterized by disruption of blood vessel walls and infiltration of leukocytes, particularly neutrophils [5]. Leukocytoclasia is the degranulation of neutrophils followed by cell death and rupture, releasing nuclear dust. LCV has a diverse etiology and may be triggered by infections, drugs, autoimmune diseases, or malignancies (Figure 3) [6].

Drug-induced LCV is uncommon, and apixaban is a rare cause of LCV, with only a limited number of reported cases. We tabulated the reported cases of LCV induced by apixaban in Table 3 [3,7-11].

**Table 3:** Reported cases of apixaban-induced leukocytoclastic vasculitis M: Male; F: Female. Source: Refs. [3,7-11].

Author	Age (years)/Sex	Day to symptoms onset	Symptoms	Confirmed on biopsy	Exclusion of other causes	Management
El-Sabbagh et al. [3]	45/M	7	Itching, purpura on lower extremity	Yes	Yes	Discontinue apixaban and oral steroids
Spears et al. [7]	95/M	12	Pruritic, non-tender purpura on lower extremity	Yes	Yes	Discontinue apixaban and oral steroids
Daul et al. [9]	74/F	7	Rash on lower extremity	No	Yes	Discontinue apixaban and topical steroids
Liedke et al. [10]	58/M	7	Rash on feet and chin	Yes	Yes	Discontinue apixaban and oral steroids
Khan et al. [11]	68/F	30	Lower extremity rash	Yes	Yes	Discontinue apixaban and oral steroids
Nasir et al. [12]	62/M	10	Burning rash	Yes	Yes	Discontinue apixaban and oral steroids

LCV pathophysiology involves immune complex deposition and complement system activation, leading to neutrophil recruitment and vessel wall injury. Elevated cytokines in response to inflammatory cascade are responsible for clinical manifestations [8]. LCV mainly develops in bilateral lower extremities because of blood turbulence and increased venous pressure in lower limbs. Apixaban-induced LCV is a diagnosis of exclusion and requires a comprehensive workup [11]. Lesion biopsy is recommended when there is a strong suspicion of drug-induced LCV, and other possible etiologies must be ruled out [4]. Histopathology of lesions varies over time. The fresh lesions manifest neutrophil infiltration and fibrinoid necrosis, while the chronic lesions have lymphocyte predominance. In drug-induced cases, the lesions may have eosinophilic infiltration [5,12].

Drug-induced LCV is managed with the removal of the offending agent and supportive measures, including leg elevation, rest, and antihistamines [13]. A short course of oral steroids may also be recommended in some cases with tapering doses. In steroid-resistant cases, steroid agents, including methotrexate or azathioprine, may also be used. However, identifying and removing the offending agent are crucial for resolution [14].

## Conclusions

Although apixaban is a well-tolerated drug, apixaban-induced LCV is a hypersensitivity reaction of the drug, an adverse event that is rarely reported. Therefore, clinicians should be aware of the potential adverse event of apixaban use, particularly in the early days of drug commencement. LCV-induced apixaban has a favorable prognosis in case of timely recognition, discontinuation of the offending agent, and appropriate management. So further research is warranted to elucidate the mechanism linking apixaban use and LCV.
